# ARTEMIS: 192-week efficacy and safety of once-daily darunavir/ritonavir (DRV/r) vs lopinavir/r (LPV/r) in treatment-naïve HIV-1-infected adults

**DOI:** 10.1186/1758-2652-13-S4-P3

**Published:** 2010-11-08

**Authors:** C Orkin, E DeJesus, H Khanlou, A Stoehr, K Supparatpinyo, T Van de Casteele, E Lathouwers, S Spinosa-Guzman

**Affiliations:** 1 Barts and The London NHS Trust, London, UK; 2 Orlando Immunology Center, Orlando, USA; 3 AIDS Healthcare Foundation, Los Angeles, USA; 4 Institute for Interdisciplinary Medicine, Hamburg, Germany; 5 Chiang Mai University, Chiang Mai, Thailand; 6 Tibotec BVBA, Beerse, Belgium

## Background

ARTEMIS was a Phase III, randomised, open-label study assessing efficacy and safety of DRV/r 800/100mg qd versus LPV/r 800/200mg total daily dose (qd or bid) in treatment-naïve HIV-1-infected adults. At 96 wks, DRV/r demonstrated non-inferiority and superiority to LPV/r in virological response. Wk 192 results are reported.

## Methods

Patients stratified by baseline (BL) viral load (VL [HIV-1 RNA] < or ≥100,000 copies/mL [cpm]) and CD4 cell count (< or ≥200 cells/mm^3^) were randomised 1:1 to DRV/r qd or LPV/r. Primary efficacy parameter: non-inferiority (≤ –12%) of DRV/r to LPV/r in virological response (VL <50 cpm, ITT-TLOVR). DRV/r superiority ( ≤ 0%) was assessed if non-inferiority was demonstrated.

## Results

689 patients (30% female; mean BL VL 4.85 log_10_ cpm; median CD4 225 cells/mm^3^) were randomised. Overall, significantly more DRV/r than LPV/r patients had VL <50 cpm at Wk 192, confirming DRV/r qd non-inferiority (p<0.001) and superiority (p=0.002) (Table 1). In patients with virological failure (VF; TLOVR non-VF censored) no developing primary PI mutations were identified in either arm; all VFs with paired BL/endpoint phenotypes that were susceptible at BL to amprenavir, atazanavir, indinavir, lopinavir, saquinavir or tipranavir remained susceptible after treatment.

**Table 1:** 

	DRV/r		LPV/r		DRV/r-LPV/r [95% CI]
	**N**	**%**	**N**	**%**	

**VL <50cpm (ITT-TLOVR)**					

All patients	343	68.8	346	57.2	11.6 [4.4-18.8]

BL VL <100,000	226	69.5	226	60.2	9.3 [0.5-18.1]

BL VL ≥100,000	117	67.5	120	51.7	15.9 [3.5-28.3]

BL CD4 <200	141	65.2	148	54.1	11.2 [-0.1-22.5]

BL CD4 ≥200	202	71.3	198	59.6	11.7 [2.4-21.0]

**VL <50cpm (sensitivity analyses)**					

TLOVR non-VF censored	270	87.4	245	80.8	6.6 [0.3-12.9]

On protocol TLOVR	340	69.1	345	57.1	12.0 [4.8-19.2]

Missing=failure	343	68.5	346	60.1	8.4 [1.3-15.5]

FDA snapshot	343	68.5	346	59.8	8.7 [1.5-15.8]

**Treatment-emergent adverse events (AEs)**					

AEs leading to permanent stop of study medication	343	7.6	346	14.5	p=0.005*

Grade 2-4 treatment-related diarrhoea	343	5.0	346	11.3	p=0.003*

**Changes in lipid parameters, median increase mmol/L (min; max)**					

Triglycerides^‡^	254	0.1 (-5; 3)	228	0.6 (-3; 10)	p<0.0001^¶^

Total cholesterol^§^	254	0.6 (-2; 4)	228	1.0 (-1; 4)	p<0.0001^¶^

*Fisher's Exact test; ^‡^NCEP normal level <1.69mmol/L; ^§^NCEP normal level <5.17mmol/L; ^¶^Wilcoxon Rank Sum test

## Conclusions

DRV/r qd demonstrated sustained efficacy with non-inferiority and superiority to LPV/r over 192 wks. Development of resistance was low in both arms. DRV/r was associated with smaller median increases in total cholesterol and triglycerides than LPV/r, and a lower incidence of grade 2–4 diarrhoea.

